# Effects of Proteases from Pineapple and Papaya on Protein Digestive Capacity and Gut Microbiota in Healthy C57BL/6 Mice and Dose-Manner Response on Mucosal Permeability in Human Reconstructed Intestinal 3D Tissue Model

**DOI:** 10.3390/metabo12111027

**Published:** 2022-10-26

**Authors:** Olha Kostiuchenko, Nadiia Kravchenko, Jan Markus, Stephen Burleigh, Olexandr Fedkiv, Ling Cao, Silvia Letasiova, Galyna Skibo, Frida Fåk Hållenius, Olena Prykhodko

**Affiliations:** 1 Department of Food Technology, Engineering and Nutrition, Lund University, 221 00 Lund, Sweden; 2 Department of Cytology, Bogomoletz Institute of Physiology, 010 24 Kyiv, Ukraine; 3 MatTek In Vitro Life Science Laboratories, 821 05 Bratislava, Slovakia; 4 Centre of Toxicology and Health Safety, National Institute of Public Health, 100 00 Prague, Czech Republic

**Keywords:** bromelain, papain, trypsin, *Akkermansia muciniphila*, intestinal integrity

## Abstract

Cysteine proteases obtained from the stem of pineapple or papaya latex, bromelain and papain, respectively, exhibit a broad spectrum of beneficial effects on human health. However, their effects on gut microbiota composition or dose-manner effects on the intestinal integrity of healthy tissue have not been evaluated. In this study, C57BL/6 young, healthy mice were fed bromelain or papain in a dose of 1 mg per animal/day for three consecutive days, followed by the assessment of digestive protein capacity, intestinal morphology and gut microbiota composition. Furthermore, a human reconstructed 3D tissue model EpiIntestinal (SMI-100) was used to study the effects of 1, 0.1 and 10 mg/mL doses of each enzyme on tissue integrity and mucosal permeability using TEER measurements and passage of Lucifer Yellow marker from the apical to the basolateral side of the mucosa. The results indicated that fruit proteases have the potential to modulate gut microbiota with decreasing abundance of Proteobacteria and increasing beneficial *Akkermansia muciniphila*. The enhancement of pancreatic trypsin was observed in bromelain and papain supplementation, while bromelain also increased the thickness of the ileal mucosa. Furthermore, an in vitro study showed a dose-dependent interruption in epithelial integrity, which resulted in increased paracellular permeability by the highest doses of enzymes. These findings define bromelain and papain as promising enzymatic supplementation for controlled enhancement of paracellular uptake when needed, together with beneficial effects on the gut microbiota.

## 1. Introduction

Cysteine proteases are identified as a large group of enzymes essential in various biological processes and can be found in microorganisms, plants, animals and even viruses [1,2]. Bromelain (EC 3.4.22.32) is a cysteine protease isolated from pineapple (*Ananas comosus* L.), while papain (EC 3.4.22.2) is obtained from papaya latex (*Carica papaya* L). Due to their antibacterial, antifungal, anti-inflammatory, antithrombotic, anticancer, fibrinolytic, and immunomodulatory properties, these two enzymes have found numerous applications in medicine as digestive assistance, as a potential adjunct in cancer therapy, in the treatment of osteoarthritis, diarrhea, sinusitis, sports injuries and respiratory tract diseases (as a mucolytic), as well as in food production, textile industry, and cosmetics [3,4,5,6]. Recently, bromelain has also been proposed as an antiviral agent against COVID-19 due to the inhibition of different variants of SARS-CoV-2 [7].

A person can consume about 12 g/day of bromelain without any noticeable side effects [8]. An in vitro study confirmed bromelain enzyme stability in artificial stomach juice and in artificial blood after 4 h [9]. In a clinical study, oral bromelain was detected to retain its proteolytic activity in plasma and was also found to be linked with blood protease inhibitors [8]. However, another study highlighted that bromelain could be partially digested, so there is the necessity to pack the protease into an acid-stable envelope or use a more refined delivery system [10].

In vitro and in vivo findings indicate that fruit protease from pineapple can improve intestinal dysmotility after postoperative or LPS-induced ileus by inhibiting colonic inducible nitric oxide synthase (iNOS) overexpression [11]. Bromelain has been successfully administered in combination therapy to alleviate symptoms of pancreatic insufficiency and dyspepsia and to enhance protein utilization in elderly patients on tube feeding [12,13].

The combination of ox bile, pancreatin, and bromelain was proven to be effective in lowering stool fat excretion in patients with pancreatic steatorrhea, resulting in symptomatic improvements in pain, flatulence, and stool frequency [12]. Applications of papain in medicine over the last five years include the treatment of proteinaceous esophageal food impaction, cure of mild and moderate acne and tissue repair of venous ulcers employing low-concentration papain gels [14].

The influence of bromelain and papain consumption on pancreatic protein digestion, the intestinal barrier and microbiota is ambiguous and have not yet been elucidated. Some studies have described the antibacterial activity of papain, and other papaya extracts against such enteropathogens as *Bacillus subtilis*, *Enterobacter cloacae*, *Escherichia coli*, *Listeria monocytogenes*, *Salmonella typhimurium*, *Staphylococcus aureus* and *Proteus vulgaris*, where plant enzymes were used to enhance food product safety [15,16]. Moreover, bromelain was shown to inhibit the growth of *Porphyromonas gingvalis*, which causes gingivitis [17].

Exogenous proteases can also influence receptors on microbiota, altering communications with their host. For instance, it has been shown that orally administered exogenous protease inhibited the activity of the K88+ enterotoxigenic *Escherichia coli* receptor and, therefore, bacterial attachment to the small intestine [18]. Several studies indicated that bromelain supplementation improved nutrient digestibility, promoted the growth of *Bifidobacterium* and *Lactobacillus*, and increased concentrations of various fecal short-chain fatty acids (SCFAs) [19,20].

Notably, although numerous studies have been conducted regarding bromelain and papain, there are limited papers that document the effect of these fruit enzymes on healthy gut microbiota and its interplay with protein digestion capacity and effects on the intestine barrier.

The present study aimed to examine separately the effects of bromelain and papain on protein digestion in relation to intestinal structure and microbiota composition in a murine model, as well as study dose-dependent effects of these fruit proteases on intestinal permeability and cell integrity using the human reconstructed 3D tissue model EpiIntestinal (SMI-100).

## 2. Materials and Methods

### 2.1. Animals

The experiment was approved by the local Malmö–Lund Ethical Review Committee for Animal Experimentation and conducted in accordance with the European Community regulation concerning the protection of experimental animals (2010/63/EU). The study called the Bromelain experiment was carried out on 13 male mice, while the study called the Papain experiment was carried out using 14 male mice of the C57BL/6 inbred strain. Animals for the Bromelain experiment were bred at the Dept facility, while for the Papain experiment, they were purchased directly from Taconic A/S (Denmark).

### 2.2. Animal Experiment Design

All animal experiments were performed at the Dept. of Biology, Lund University at 20 ± 1 °C, 50 ± 10 RH%, 12:12 h light/dark cycle, using young mice at least two weeks after weaning (6–8 weeks of age). Mice were kept in polycarbonate cages with aspen wood bedding (Beekay B&K Universal AB) and supplemented with paper-nesting material (Sizzle-pet; Lillicobiotech) with free access to water, and a rodent laboratory chow (R3, Lactamin) placed on the cage lid. 

Since animals were of the same strain but from different breeders, each experimental set included its own control. Thus, animals in the Bromelain experiment were divided into 2 groups and fed once a day for 3 consecutive days with either bromelain (BRM, n = 7) or water (CRTL, n = 6). In comparison, animals in the Papain experiment were first acclimatized for one week to the Dept Animal facility and then divided into 2 groups and fed once a day for three consecutive days with either papain (PAP, n = 7) or water (CTRL, n = 7) via soft feeding tubes (Supplementary Figure S1). 

Bromelain, EC 3.4.22.32; CAS 37189-34-7, and papain, EC 3.4.22.2; CAS 9001-73-4, were purchased from BioChemica (AppliChem, St. Louis, Missouri, USA). A single dose for feeding was calculated as 1mg per mouse and made by dissolving enzyme powder in distilled water (10 mg/mL, feeding volume 0.1 mL), which is equivalent to the total protease activity measured in 250 mL of freshly prepared pineapple juice or 100 g of papaya fruit as to be consumed by person weighing 60 kg. The control group received 0.1 mL water instead of the fruit enzyme solution. Body weight was measured daily.

### 2.3. Animal Material Collection

Twenty-four hours after the last administered dose, the animals were weighed, anesthetized by inhalation of Isoflurane (Baxter Medical AB, Kista, Sweden) and sacrificed by opening the thorax and exsanguination via direct heart puncture. The intestines were dissected out, weighed and separated into duodenum, jejunum, ileum and upper colon samples, which were stored in 4% paraformaldehyde for further histological evaluation. The pancreas gland and cecum were immediately frozen for protease activity measurements. A piece of the cecum containing both tissue and cecal content was cut out in aseptic conditions and frozen at −80 °C for the next-generation sequencing analysis of microbiota.

### 2.4. EpiIntestinal Human Tissue Model (SMI-100)

**Figure 1:**
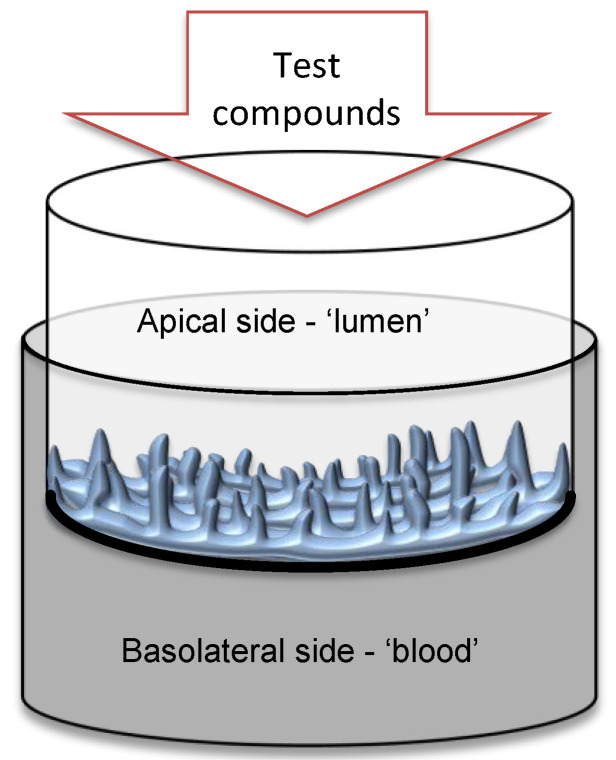
Scheme of the EpiIntestinal 3D human tissue cultivation in the insert with polycarbonate semipermeable membrane. Enterocytes face the apical side ´lumen,’ while the basolateral side of the epithelium faces the ´blood´ medium.

Thirty-six in vitro reconstructed human tissues (EpiIntestinal^TM^ SMI-100; Figure 1) from young adult healthy donors were purchased from MatTek In Vitro Life Science Laboratories, Bratislava, Slovak Republic. First, tissues were pre-equilibrated overnight to air-liquid conditions with SMI-100-MM feeding medium (MatTek) at 37 °C, 5% CO_2_ and 95% RH. The experiment was performed the following morning after washing the tissues with SMI-100-MM medium from the mucus and shedding cell debris.

### 2.5. Tissue Experimental Design

Flat bottom untreated Falcon^®^ 24-well polystyrene plates (Fisher Scientific, Hampton, NH, USA) were filled with 0.5 mL of pre-heated basolateral/receiving buffer (Hank’s Balanced Salt Solution (HBSS, Gibco, Grand Island, NY, USA) with addition of 0.2% glucose (Sigma, St. Louis, MO, USA) and 0.01M HEPES (Sigma), pH 7.4. Bromelain or papain powder was dissolved in apical/donor buffer HBSS with glucose and HEPES, pH 6.5 at the concentration of 10 mg/mL, immediately filtered through 0.2 μm syringe filter and further diluted to 1 mg/mL and 0.1 mg/mL dose. HBSS solution containing no bromelain or papain was used as a control. Tissues were treated by applying 0.1 mL of each apical buffer as follows: BRM_10 (n = 6), BRM_1 (n = 6), BRM_0.1 (n = 6), PAP_10 (n = 4), PAP_1 (n = 4), PAP_0.1 (n = 4) and CTRL (n = 6). All groups were incubated for 30 min at 37 °C, 5% CO_2_. After incubation, the apical buffer was removed, tissues were washed with apical/donor buffer HBSS, and Lucifer Yellow (LY) fluorescent marker in a volume of 0.1 mL and a concentration of 100 μM was applied on the apical surface. After an additional 30 min incubation, each sample’s apical and basolateral solutions were collected to estimate LY passage while tissues were fixed in 4% formaldehyde for further histological evaluation.

### 2.6. TEER Measurement in the Tissue Model

Trans-epithelial electrical resistance (TEER) assay was performed before the treatment (at 0 min), directly after the treatment (at 30 min), and after the washout period (at 60 min) to evaluate the tissue integrity. In brief, tissues were washed with sterile 100 mM KCl, and then TEER was measured in individual tissue inserts using the EVOM2^TM^ Epithelial Voltohmmeter and ENDOHM-12 tissue resistance measurement chamber (World Precision Instruments, Sarasota, FL, USA). This was done in 2 repetitions for each tissue sample and for each time point. Next, the resistance was recalculated to the surface area of the tissue and presented as Unit area resistance, Ω*cm^2^.

### 2.7. MTT Assay

To measure cell viability, 3 tissue inserts were incubated with 100 μL of the highest enzyme dose of papain or bromelain (10 mg/mL), two with PBS (negative control) and 2 with 0.3% Triton X100 (positive control for the method) at 37 °C, 5% CO_2_ and 95% RH for 1 h. Next, after extensively washing with 8–10 changes of PBS, the cellular oxidoreductase enzymes were measured to assess cell viability by estimating the reduction of the tetrazolium dye MTT to its water-insoluble form formazan during 3 h at 37 °C, 5% CO_2_. Next, formazan was extracted by overnight incubation with isopropanol at room temperature and measured using a spectrophotometer at 570 nm [21].

The result was recalculated as follows:

% viability = [OD_570_ (test)/OD_570_ (negative control)] × 100

### 2.8. Passage of Lucifer Yellow (LY)

Passage of a 452 Da fluorescent marker from the ‘luminal’ to ‘blood’ side was estimated in apical and basolateral liquids collected at 1 h after the initial experimental point, which coincided with the end of a 30-min washout period. LY emission was measured using a Fluoroskan microplate reader (Thermo Fisher Scientific, Waltham, MA, USA) and the appropriate filter set (Ex 485 nm, Em 538 nm). A series of LY dilutions with known concentrations was used as a standard.

Since the surface area of all tissues was equal, results were recalculated as a percentage of LY penetrated through the tissue showing intestinal uptake using the following simplified formulas [22]:

% uptake = 100 − % rejection

% rejection = (1 − [C_basolateral_]/[C_apical_]) × 100

### 2.9. Histology and Microscopy

After fixation, all tissue samples were washed with PBS and embedded into OCT cryo-molds (SAKURA) using TissueTek^®^ (HistoLab, Gothenburg, Sweden). The cryosectioning of tissues was performed on Leica CM1860 Cryostat (Leica Microsystems AB, Stockholm, Sweden). Six-μm thin tissue sections were mounted onto polylysine-coated adhesion slides (TermoFisher Scientific, Waltham, MA, USA), dried for 1 h at room temperature and stored at −20 °C. Hematoxylin and Eosin (H&E) staining was performed according to the standard protocol, and tissues were mounted under coverslips and evaluated using an Olympus microscope BX60 (Olympus Optical Co, Tokyo, Japan). Images of tissues for morphometric analyses were taken using an Olympus DP74 camera, and the measurement of mucosal thickness was performed using ImageJ software (NIH, Bethesda, Maryland, USA). Mucosal thickness was defined as the distance from the tip of the villus to the base of the muscularis mucosa [23]. The measurement of the mucosal thickness of each sample was repeated at least 10 times from different slices, and the average for each animal was determined.

In addition, reconstructed tissue samples exposed to LY fluorescent marker were washed in PBS to remove the OCT compound, mounted under a coverslip with PBS and glycerol (1:1) and microscopically analyzed for fluorescent emission. Mucosal thickness after administration of bromelain with different concentrations was also assessed, as described above.

### 2.10. Gut Microbiome Sequencing

Extraction of DNA was performed from 50–100 mg of cecal content using the QIAamp DNA Stool Mini Kit (Qiagen, Hilden, Germany), according to the manufacturer’s protocol using an additional bead-beating step. Measurement of DNA concentration was performed using a Qubit 4.0 Fluorometer (Thermo Fisher Scientific, Waltham, MA, USA). The V4 regions of 16S rRNA genes were amplified using forward 515F, 5′-GTGCCAGCMGCCGCGGTAA-3′ and reverse primers 806R 5′- GGACTACHVGGGTWTCTAAT-3′ containing Illumina overhang adaptors and unique dual indexes as described by Kozich et al. [24] Paired-end sequencing with a read length of 2 × 250 bp using a MiSeq V2 reagent kit was carried out on a Miseq Instrument (Illumina Inc., San Diego, CA., USA). Sequencing data were analyzed using the open-source bioinformatics pipeline Quantitative Insights into Microbial Ecology (QIIME v1.9) [25]. In total, 1,489,545 reads were used for the 13 samples in the ‘bromelain experiment’ (BRM, n = 7; Ctrl, n = 6) with a mean of 114,580 reads per sample, (min: 41,739 and max: 185,863), while 1,588,217 reads were used for the 14 samples in the ‘papain experiment’ (PAP, n = 7; Ctrl, n = 7) with a mean of 113,444 reads per sample, (min: 72,795 and max: 142,149). 

The sequences were grouped into operational taxonomic units (OTUs) by UCLUST at a minimum of 97% sequence similarity. Representative sequences (most abundant) from each OTU were aligned using Python Nearest Alignment Space Termination (PyNAST). Taxonomy was assigned using the Greengenes database v.13.8 (http://greengenes.lbl.gov, accessed on 1 October 2018) [26]. OTU tables are available in supplement Table S1.

### 2.11. Enzymology and Protein Detection

Trypsin enzyme activity was measured in the pancreas and in cecal homogenates (1:10 wt/vol). The pancreatic homogenates were incubated with enteropeptidase to activate trypsin before incubating with a trypsin-specific substrate, benzoyl-DL-arginine-4-nitroanilide (BAPNA; Merck, Merck Life Science AB, Darmstadt, Germany f.k.a Sigma-Aldrich). The trypsin activity unit (U) was recalculated as the amount of enzyme that catalyzes 1 μmol of substrate per minute.

A universal protease activity assay was performed to estimate the total proteolytic activity in freshly squeezed juice made from pineapple or papaya fruit for dose recalculation and in murine cecal homogenates. In brief, samples were incubated with casein as a protease substrate at +37 °C for 10 min while the quantity of liberated amino acid tyrosine during this time was measured after reacting with Folin’s reagent according to manufacturer technical protocol (Merck, Merck Life Science AB, Darmstadt, Germany) [27]. L-tyrosine with known concentrations was used for standard curve preparation. 

The protein concentration in the supernatants was determined using the Pierce Coomassie (Bradford) Protein assay with serum albumin as a standard (Thermo Fisher Scientific, Waltham, MA, USA). The activity of enzymes was recalculated per mg of total protein.

### 2.12. Statistical Analysis

The results are presented as mean ± SEM. Statistical analyses, calculations, Pearson correlation and graph preparations were done using Prism 9 (GraphPad software, San Diego, CA, USA). For the 2 groups, Student’s unpaired t-test was performed, while for the comparison of the 4 groups tested in vitro model, a one-way ANOVA and Tukey’s multiple comparison posthoc test were performed. The differences were considered to be statistically significant when *p* < 0.05, and a trend was discussed when *p* ≤ 0.1. In addition, the Pearson correlation test for total protease activity to total protein in cecum has been performed for the whole cohort of studied animals (27 of XY pairs).

## 3. Results

### 3.1. In Vivo Study

Neither body weight gain nor the weights of the small intestine, cecum and large intestine were affected by bromelain or papain gavage during the three-day treatment in mice. The data are available in a (supplement Table S2).

#### 3.1.1. Histological Examination and Morphometric Analysis of the Intestinal Tissue

**Figure 2:**
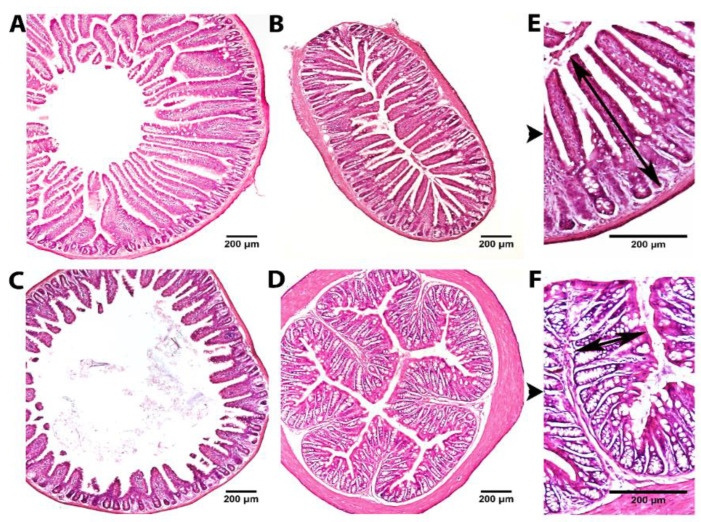
Representative images of H&E staining of the duodenum (**A**), jejunum (**B**), ileum (**C**) and upper colon (**D**) at ×40 magnification and jejunum (**E**) and upper colon (**F**) at ×100 magnification. Black arrows mark the mucosal thickness measurement.

To evaluate the effect of the two fruit proteases on intestinal mucosal morphology, the different portions of the small (duodenum, jejunum, ileum) and large (upper colon) intestines were examined after H&E staining using light microscopy. Histological analysis indicated a normal pattern with intact and well-packed intestinal villi in all control and treatment groups (Figure 2). The results of the morphometrical study comparing the mucosal thickness of controls, bromelain and papain groups are shown in Table 1. After bromelain administration, the thickness of mucosa in the ileum significantly increased in comparison to the control group (*p* < 0.05). Moreover, duodenal mucosal thickness tended to increase as well in the group with bromelain supplementation (*p* = 0.1). Papain treatment had no effect on mucosal thickness parameters in all intestine parts. Noteworthy, there was a significant difference detected between the two control groups in relation to the thickness of the mucosa in the jejunum and colon, which might be explained by variations in animal breeding and litter. 

#### 3.1.2. Microbiota Composition Analysis

Sequenced data of the V4 16S RNA gene showed normal distribution of samples for both bromelain and papain experiments evaluated by the Shapiro–Wilk normality test. The general spreading of the cecal microbiota data after the principal component analysis resulted in the clustering of enzyme-treated groups vs. water, higher alpha diversity for BRM and PAP group (Chao1, Shannon) although not significant (Figure S2). 

**Figure 3:**
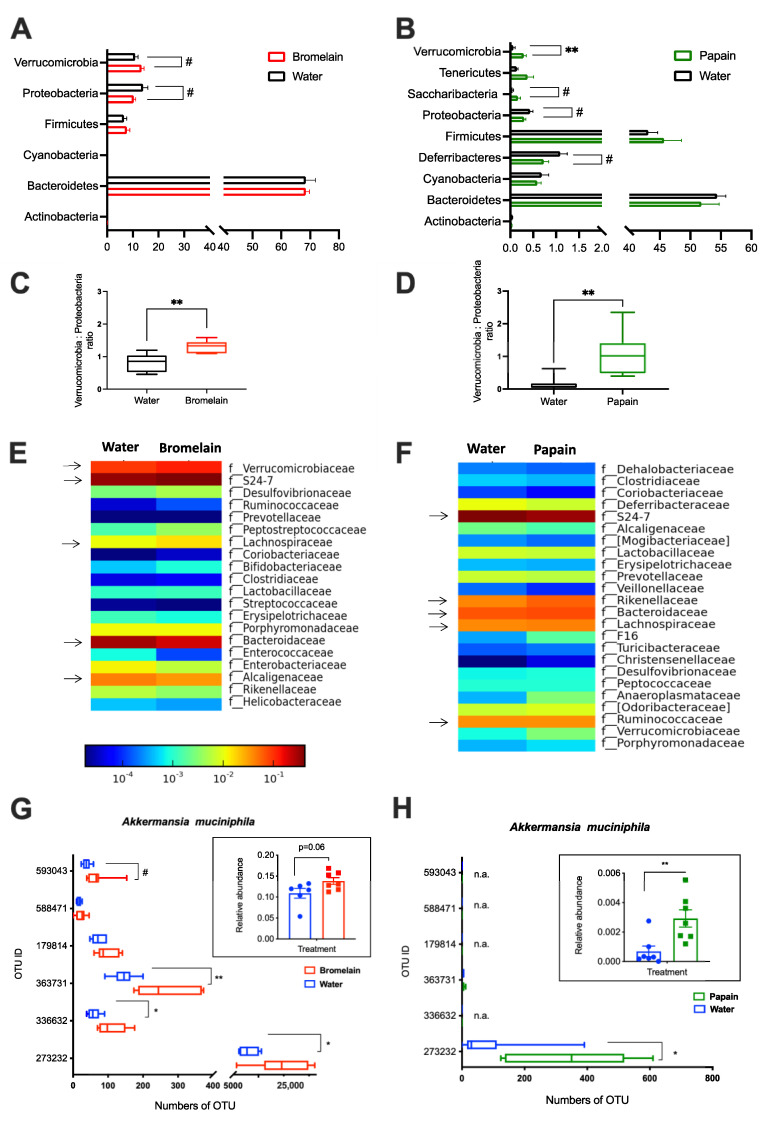
Composition of the gut microbiota in mice from *Bromelain* (**A**,**C**,**E**,**G**) and *Papain* experiments (**B**,**D**,**F**,**H**). Bar plots show the relative abundance of cecal bacterial community at Phylum level (**A**,**B**); the ratio between Verrucomicrobia to Proteobacteria (**C**,**D**); Family level (**E**,**F**) and amount of OTU ID in each experimental group which has been assigned to *Akkermansia muciniphila* (**G**,**H**). Data are shown as mean ± SEM, (n = 6–7 per group) * *p* < 0.05, ** *p* < 0.01, # *p* < 0.1. Arrows indicate the five most abundant families.

Bromelain experiment: The grouping of bacterial sequences into OTUs resulted in six bacterial phyla, Bacteroidetes, Proteobacteria, Verrucomicrobia, Firmicutes, Actinobacteria, and Cyanobacteria (Figure 3A). The bacterial community at the phylum level was not significantly changed in bromelain-fed mice, but the Proteobacteria phylum showed a decrease (13.6% CTRL vs. 9.9% BRM, *p* < 0.1) due to the lowered abundance of such classes as Betaproteobacteria (from 4.9 to 3.8%), Epsilonproteobacteria (from 6.6 to 4.3%), Gammaproteobacteria (from 1.4 to 0.7%) as compared to water-fed control. The abundance of Alphaproteobacteria was not changed (0.1% for each group), while Deltaproteobacteria was significantly increased after bromelain feeding (from 0.5% to 1%, *p* < 0.05). Verrucomicrobia phylum showed a tendency to increase (10.2% CTRL vs. 13.5% BRM, *p* = 0.06) compared to control. Furthermore, the Verrucomicrobia to Proteobacteria ratio was found to be significantly higher in the bromelain group compared to the water-fed control (*p* < 0.01; Figure 3C). At the family taxonomic level, the five most abundant bacterial families were represented by *S24-7* (40.4%), *Bacteroidaceae* (27.1%), *Verrucomicrobiaceae* (12%), *Helicobacteraceae* (5.5%), *Lachnospiraceae* (5.1%) and *Alcaligenaceae* (4.3%), from which only *Verrucomicrobiaceae* showed a tendency to increase (10.2% CTRL vs. 13.5% BRM, *p* = 0.06) compared to control, although different OTU ID assigned to *Akkermansia muciniphila* showed significant differences between bromelain- and water-fed mice (Figure 3E,G).

Papain experiment: At the phylum level, the cecal microbiota in mice were presented by Bacteroidetes, Firmicutes, Deferribacteres, Cyanobacteria, Proteobacteria, Tenericutes, Verrucomicrobia, Saccharibacteria (f.k.a. TM7) and Actinobacteria. Papain feeding did not result in any significant changes in Bacteroidetes, Firmicutes, Cyanobacteria and Actinobacteria phyla. However, the relative abundance of Verrucomicrobia was significantly higher in papain-fed mice compared to the water-fed control (0.29% vs. 0.07%, respectively, *p* = 0.007). There was also a tendency for an increased abundance of Saccharibacteria phyla after papain administration (0.04% CTRL vs. 0.17% PAP, *p* = 0.07. In contrast, Deferribacteres (1.1% CTRL vs. 0.7% PAP, *p* = 0.08) and Proteobacteria (0.43% CTRL vs. 0.3% PAP, *p* = 0.09) tended to decrease in papain-fed mice due to decreased relative abundance of such classes as Betaproteobacteria (from 0.24% to 0.15%) and Deltaproteobacteria (from 0.2% to 0.15%). Furthermore, the Verrucomicrobia to Proteobacteria ratio was found to be significantly higher in the papain group compared to the water-fed control (*p* < 0.01) (Figure 3D). At the family taxonomic level in the *Papain experiment*, the five most abundant bacterial families were represented by *S24-7* (32%), *Ruminococcaceae* (11.2%), *Bacteroidaceae* (8%), *Lachnospiraceae* (6%) and *Rikenellaceae* (6%). The relative abundance of *Verrucomicrobiaceae*, as well as the *Peptococcaceae* family, was significantly increased in the PAP group compared to the water-fed control (*p* < 0.01). In comparison to the control group, mice fed with papain showed an increase in the relative abundances of the unclassified family from *Bacteroidales* (0.32% CTRL vs. 4.26% PAP, *p* < 0.001) and unclassified genus from *Rikenellaceae* (4.06% CTRL vs. 5.98% PAP, *p* < 0.05), as well as a decrease in the unclassified genus from *S24–7* (41.63% CTRL vs. 32.08 PAP, *p* < 0.05) family. Papain-fed mice showed an increased abundance in the genus *Akkermansia* (0.07% CTRL vs. 0.29% PAP, *p* < 0.01) and were assigned mainly by OTU ID 273232 (Figure 3 B,D,F,H).

#### 3.1.3. Effect of Plant Enzymes on Protein-Digestive Properties 

**Figure 4:**
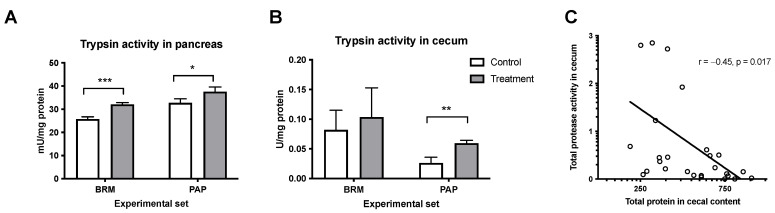
Effect of bromelain and papain on the activity of trypsin in the pancreas (**A**) and cecum (**B**). A t-test was used, and the Pearson correlation test was used to determine the total protease activity to total protein in the cecum (**C**). Significant differences denoted by * *p* < 0.05, ** *p* < 0.01 *** *p* < 0.001. Values are represented as mean ± SEM for n = 6–7 per group.

Trypsin is a digestive enzyme, and its activity is often monitored as an important indicator of metabolic rate [28]. The trypsin activity was analyzed in the pancreas and in the cecum after bromelain and papain treatments. Treatment with plant enzymes resulted in a significant elevation of trypsin activity in the pancreas of animals from the respective experimental groups (Figure 4A). On the other hand, only papain treatment had a significant effect on endogenous protease activity in the cecum (Figure 4B), although the tendency to increase the activity can be observed in the bromelain group. Additionally, the correlation analysis showed negative relation (r = −0.45, *p* = 0.017) between total protease activity in the cecum to total protein in cecal content in the studied cohort of animals (Figure 4C).

### 3.2. In Vitro Study

#### 3.2.1. Intestinal Integrity

**Figure 5:**
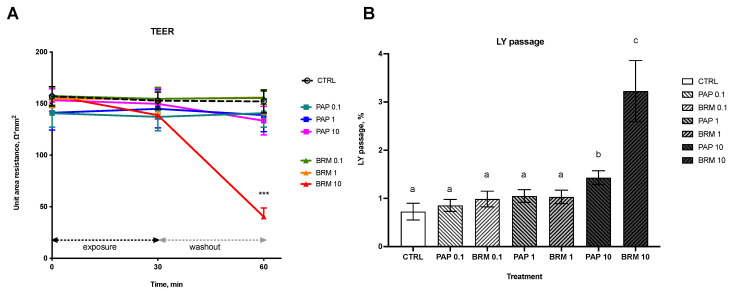
Bromelain and papain effect on the epithelial integrity (**A**) as measured by TEER and permeability for LY marker (**B**). Significant differences denoted by *** *p* < 0.001 (for **A**) and with different letters–groups not sharing a common letter are significantly different from each other; *p* < 0.05 (for **B**).

The TEER assay showed a significant decrease of area resistance, Ω*cm^2^, at 60 min for the BRM_10 group as compared to CTRL (40.41 ± 8.45 vs. 152.17 ± 11.16, respectively, *p* < 0.001), which indicated decreased tissue integrity (Figure 5A). Other groups after BRM and PAP administration with different concentrations and at different time points displayed no significant difference in area resistance compared to the control.

#### 3.2.2. Intestinal Permeability and Cell Viability

The effect of fruit enzymes on intestinal paracellular uptake of low-weight molecules was studied using an LY passage assay. Only treatment with the highest concentration (10 mg/mL) of both bromelain and papain resulted in a significant increase in % LY passage from apical to basolateral side (*p* < 0.05; BRM_10 0.73 ± 0.18 n = 6; PAP_10 1.43 ± 0.14 n = 6 groups vs. non-treated control, CTRL 3.23 ± 0.63, n = 6). Lower concentrations of PAP and BRM (1 mg/mL and 0.1 mg/mL) did not cause any significant changes in LY uptake (Figure 5B).

Noteworthy, tissue viability was unaffected by PAP_10 or BRM_10 after 1 h exposure period as evaluated by MTT assay. Viabilities were 94.40 ± 3.34% for PAP_10, 92.89 ± 4.55% for BRM_10. Treatment with Triton X-100 control resulted in 33.71 ± 4.18% cell viability.

#### 3.2.3. Histology of Reconstructed Tissues

**Figure 6:**
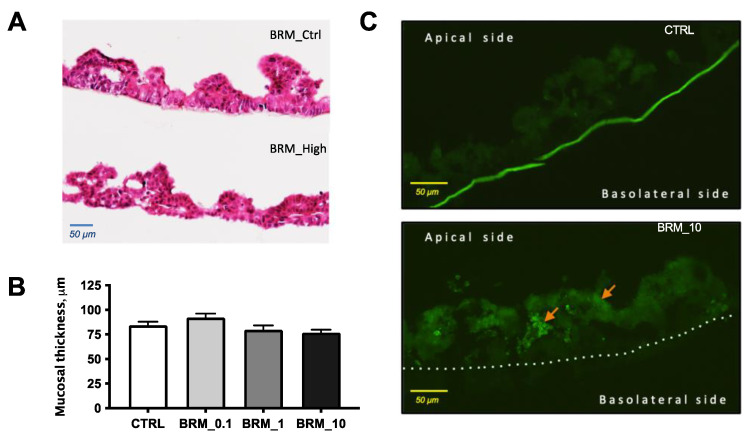
Representative photomicrographs of H&E-stained tissue samples from the untreated control (**upper**) and treated with 10 mg/mL BRM (**lower**) (**A**); mucosal thickness measured from the tip of villi-like structure to the membrane (**B**); photomicrographs of fluorescent samples from untreated control (**upper**) and treated samples with 10 mg/mL BRM (**lower**) showing presence LY marker in the mucosa by arrows (**C**).

Morphometric evaluation of H&E-stained tissue samples (Figure 6A) from in vitro study showed no effect of enzyme treatment on mucosal thickness (Figure 6B), while fluorescent microscopy evaluation showed the presence of LY in the mucosa treated with the highest dose of bromelain (Figure 6C).

## 4. Discussion

There is growing evidence that fruit proteases can be used as efficacious dietary supplements to prevent inflammatory bowel disease, colitis, diabetes, cancer and various cardiovascular diseases [29,30]. However, the mode of their action is not yet well understood. The present study was undertaken to ascertain the effect of two fruit proteases, bromelain and papain, on gut morphology and microbiota composition, given that the intestine is the first and main place of action for dietary enzymes to exhibit their biological properties.

Histological evaluation of the different structural parts of the small and large intestine in mice revealed that bromelain supplementation significantly increased the mucosal thickness in the ileum, which could indirectly indicate the enhanced proliferation of the stem cells of the intestinal mucosa. This observation is in line with a previously published study showing increased intestinal cell renewal after protease ingestion in rats [31]. Several studies stated that improvement of mucosal thickness coincides with greater absorption capacity [32,33,34], and thus, we could assume that bromelain treatment might enhance intestinal absorption not only for drugs, as it has been reported, but also for the nutrients which need to be further explored.

Various nutritional interventions were shown to modulate host gut microbiota crosstalk as well as shape the gut microbial community through the supply of substrates for the metabolic requirements of individual microbial taxa [35]. To our knowledge, only a few studies reported that bromelain and pineapple stem flour could promote the growth of *Lactobacillus* spp. and *Bifidobacterium* spp. [20,36]. However, no prior research has examined the influence of papain and bromelain on healthy gut microbiota. In our study, supplementation with bromelain and papain induced changes in the cecal microbiota of the young mice, namely a notable shift in the Verrucomicrobia and Proteobacteria phyla, resulting in a remarkable increase of Verrucomicrobia to Proteobacteria ratio. This ratio may be further used in the estimation of health-benefit effects, for instance, in nutritional interventions. Since a number of studies reported that an increased abundance of Proteobacteria is associated with metabolic disorders and gut inflammation [37,38,39], while a decrease in Verrucomicrobia has been associated with impaired intestinal barrier function, obesity, and insulin resistance [40,41]. Intriguingly enough, our findings indicate that both fruit proteases promoted an increase of health-beneficial *Akkermansia muciniphila*, a member of the Verrucomicrobia, colonizing the intestinal mucosa of rodents and humans. It was previously described that the high relative abundance of *A. muciniphila* positively correlated with the level of mucins in the cecum of rodents, leading to an increase in mucus thickness and enhancement of the gut barrier [42]. Although *A. muciniphila* is a mucin-degrading bacterium, it is also found to stimulate mucin production along with an increase in expression of the epithelial tight junction proteins (occludin and ZO-1), thus improving gut barrier function [43,44,45]. The present study indicates that the increased relative abundance of *Akkermansia* is induced by the administration of proteases from papaya and pineapple and could be of importance for promoting intestinal health since the microbial colonization of the mucosal layer by *A. muciniphila* can potentially modulate the gut environment. 

Since oral proteases have been shown to contribute to the protein digestion process *per se*, as well as via stimulation of pancreatic function [31], pancreatic trypsin production, as well as active trypsin in the cecum, were evaluated. The results revealed that both plant enzymes significantly increased trypsin activity in the pancreas, presumably via enhanced bioavailability of amino-acids liberated from dietary protein [46] or via direct stimuli on mucosal receptors, since bromelain enzyme can survive luminal digestion as shown in mice [47]. Moreover, papain boosted the presence of active trypsin in the cecum, which was accompanied by reduced protein content in the chyme. Thus, it is assumed that a plant enzyme-enriched diet could influence the hydrolysis of proteins into short-chain peptides, which increases food digestibility and modifies gut microbiota composition. Previous studies have reported that bromelain supplementation increased the total tract digestibility of nutrients in pigs, rats and chickens, while a papain-enriched diet could speed up the digestive process of fish [19,48]. Our findings are also in line with published research, which suggested that the supplementation of pancreatic digestive enzymes induces colonization of *A. muciniphila* [49]. Therefore, we presume that fruit proteases have the potential to alter the gut microbiome by enhancing protein-digestive capacity, providing substrates for bacterial metabolic requirements.

The present study also aimed to confirm the direct effects of fruit cysteine proteases on intestinal epithelial integrity and permeability for low molecular weight markers using a human reconstructed tissue model. Since it has been previously shown that intestinal uptake via paracellular route is a promising target for non-protein drug delivery, several studies reported that dietary enzymes might enhance intestinal paracellular uptake of low-weight molecules as shown in vivo, *post-vivo* and in vitro in Caco-2 cancer cell line [50,51]. The present study introduced the novel, non-cancer, animal-free approach [52] to studying the effects of dietary enzymes on tissue integrity and molecular uptake by using human-reconstructed 3D intestinal tissues. Here, using the LY passage assay, we showed that the effects of dietary enzymes are dose-depended, where only the highest concentration of bromelain and papain (10 mg/mL) significantly increased passage of LY from the ’lumen’ to the ’blood’ side. These results are consistent with findings where bromelain has been shown to increase drug uptake by making intestinal mucus more permeable, while papain was described as an effective permeation enhancer for orally administered low molecular weight heparin [51,53]. Moreover, the results from TEER measurements during the study indicated that bromelain in a concentration of 10 mg/mL significantly decreased the area resistance, thus compromising mucosal integrity. The decrease in TEER values after bromelain administration could be interpreted as self-enhanced paracellular diffusion. This hypothesis is further supported by the already known strong mucolytic activity of bromelain and papain, based upon the cleavage of amino acid binding sequences of mucus glycoproteins [54] and by their known effect on the tight junction proteins [55,56].

In conclusion, our results attempted to expand knowledge about plant proteases on gut digestive-, barrier functions and microbiota composition in healthy conditions.

Oral administration of bromelain and papain to healthy young mice demonstrated a stimulatory effect on pancreatic function, resulting in improved digestion capacity of dietary protein. The study also showed that bromelain enhances ileum thickness in experimental animals. Furthermore, both proteases were found to be favorable to beneficial *Akkermansia muciniphila*. In vitro study demonstrated that epithelial integrity might be affected by fruit enzymes in a dose-dependent manner, but effects of papain were found to be slighter on small intestinal mucosa compared to bromelain treatment of the same dose. 

## Figures and Tables

**Table 1:** Effect of fruit proteases feeding during a 3-day period on the mucosal thickness of the small and large intestines in mice.

Mucosal thickness, μm	Bromelain		Papain	
	Control	Treatment	Control	Treatment
Small intestine:				
Duodenum	643.3 ± 25.7	719.4 ± 34.5 **^#^**	601.2 ± 16.3	584.3 ± 14.9
Jejunum	458.2 ± 38.5 ^a^	453.4 ± 14.9	373.1 ± 13.9 ^b^	369.3 ± 14.3
Ileum	270.2 ± 28.8	356.2 ± 24.0 *	313.1 ± 14.3	317.1 ± 11.6
Large intestine:				
Upper colon	194.2 ± 27.8 ^a^	187.1 ± 28.8	121.3 ± 14.1 ^b^	139.7 ± 15.63

Results presented as mean ± SEM. Bromelain: control n = 6; treatment, n = 7; Papain: control n = 7; treatment n = 7. The statistical difference between control and treatment is presented by symbols, * *p* < 0.05, # *p* = 0.1; while difference between controls of experimental sets is presented by different letters, a vs. b, *p* < 0.05

## Data Availability

Data supporting reported results can be found at https://www.ncbi.nlm.nih.gov/sra/ (accessed on 1 October 2018). Projects entitled PRJNA890026 and PRJNA890035 are corresponding to Bromelain and Papain study, respectively.

## Supplementary Materials

The following supporting information can be downloaded at: https://www.mdpi.com/article/10.3390/metabo12111027/s1. Figure S1: Experimental design of the animal study; Figure S2: Alpha diversity (Chao1, Shannon), PCoA; Table S1: OTU table; Table S2: Animal studies.
