# Supplementary material for: Effects of Proteases from Pineapple and Papaya on Protein Digestive Capacity and Gut Microbiota in Healthy C57BL/6 Mice and Dose-Manner Response on Mucosal Permeability in Human Reconstructed Intestinal 3D Tissue Model
Source: Metabolites. 2022 Oct 26;12(11):1027. doi: 10.3390/metabo12111027 (PMC9696696; doi:10.3390/metabo12111027)
Supplement: Supplementary file 1 [file metabolites-12-01027-s001.zip › Figure S1_Experimental design of the animal study.pdf]

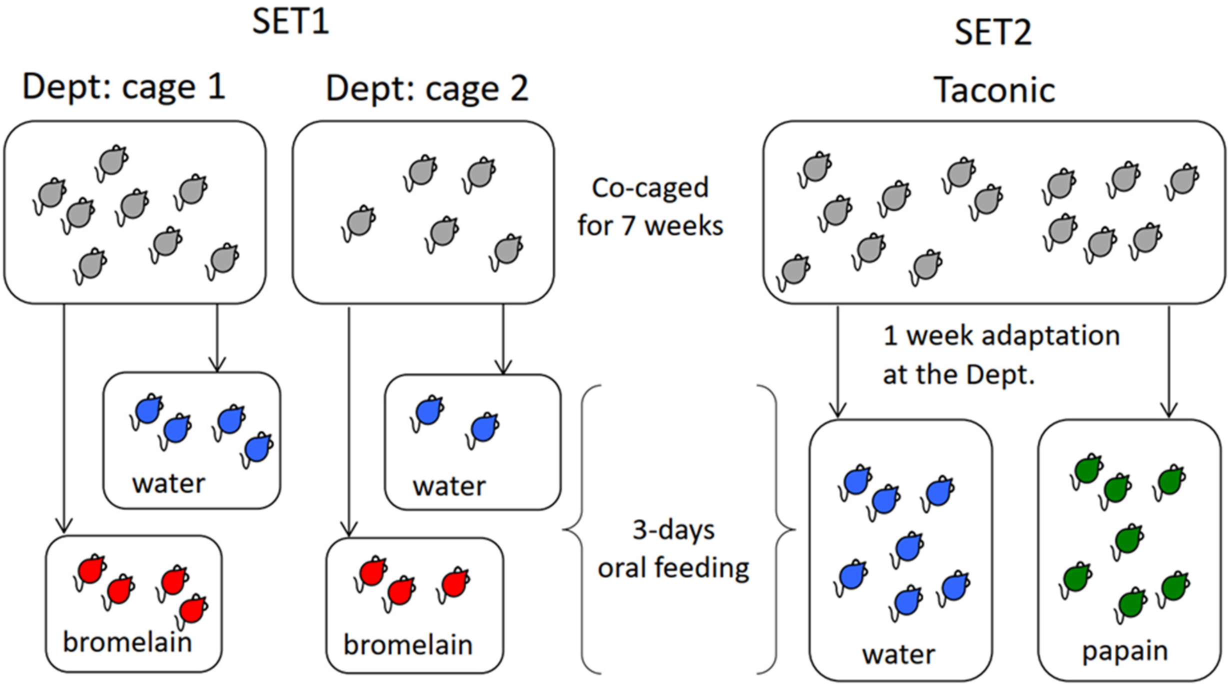

Figure S1. Experimental design of the animal study. In the current study, two different sets of animals were used. SET1 – water n = 6, bromelain n = 7 were primarily obtained from Taconic A/S, Denmark and bred in the Dept. of Biology, Lund University under specific pathogen-free conditions; SET2 – water n = 7, papain n=7 were purchased directly from Taconic. During three consecutive days, animals had oral feeding, control groups with water and experimental groups with plant proteases.
