# Supplementary figures and images for: Effects of Proteases from Pineapple and Papaya on Protein Digestive Capacity and Gut Microbiota in Healthy C57BL/6 Mice and Dose-Manner Response on Mucosal Permeability in Human Reconstructed Intestinal 3D Tissue Model
Source: Metabolites. 2022 Oct 26;12(11):1027. doi: 10.3390/metabo12111027 (PMC9696696; doi:10.3390/metabo12111027)

A

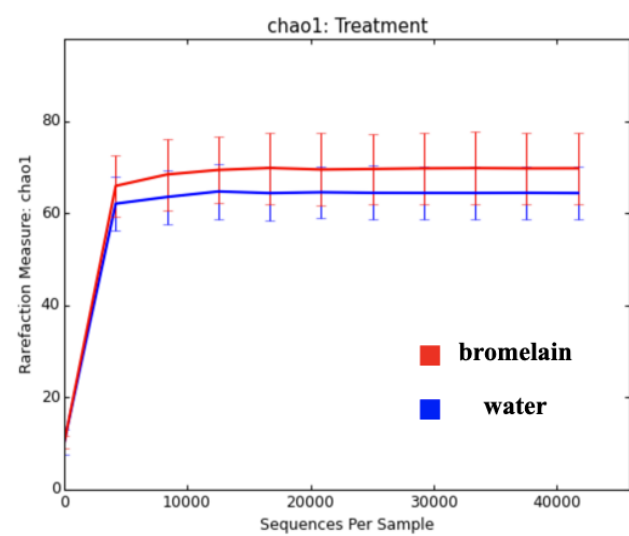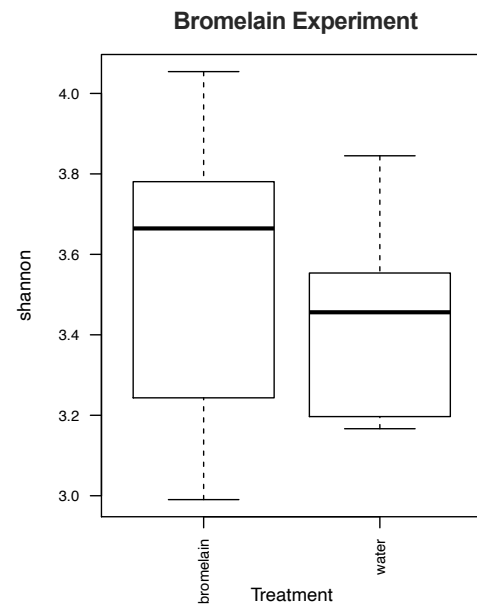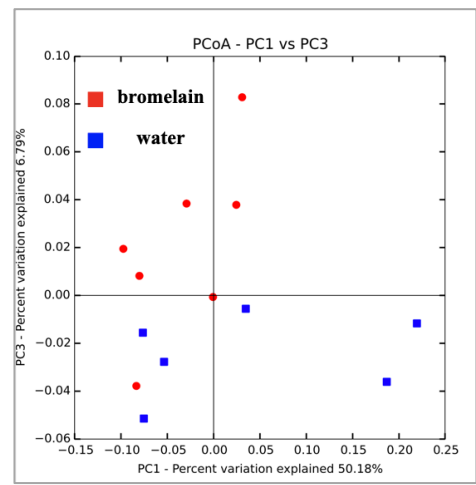

B

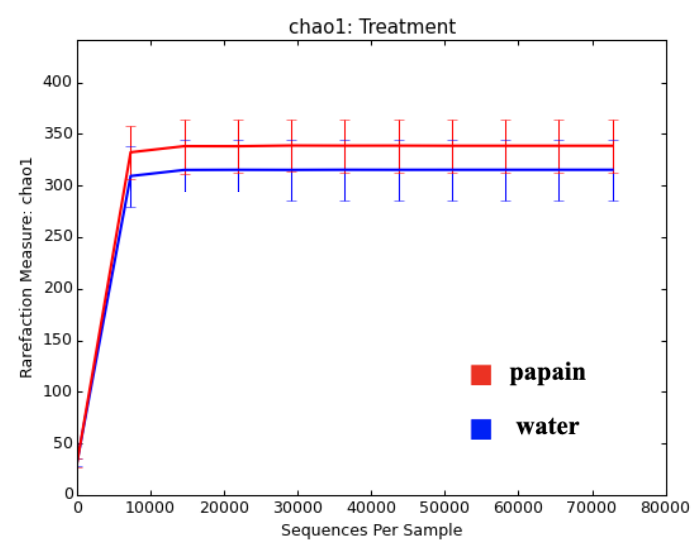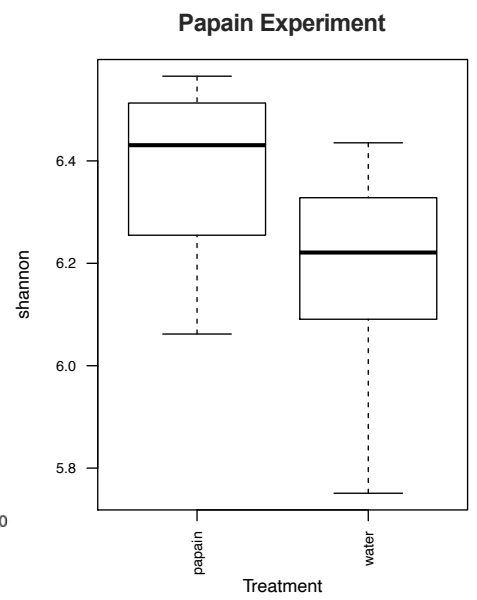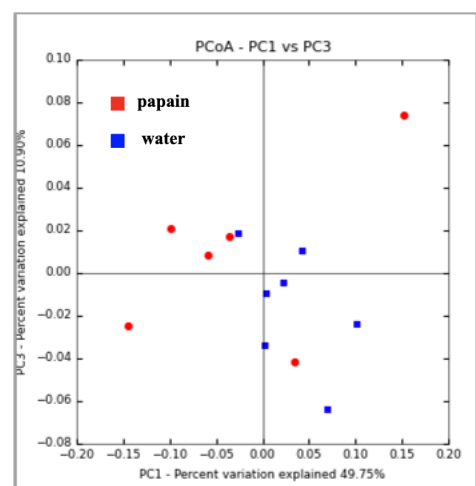

Supplement: Supplementary file 1 [file metabolites-12-01027-s001.zip › Figure S2_Alpha diversity (Chao1, Shannon), PCoA.pdf]
